# Do mobile clinics provide high-quality antenatal care? A comparison of care delivery, knowledge outcomes and perception of quality of care between fixed and mobile clinics in central Haiti

**DOI:** 10.1186/s12884-017-1546-7

**Published:** 2017-10-16

**Authors:** Erica Phillips, Rebecca J. Stoltzfus, Lesly Michaud, Gracia Lionel Fils Pierre, Francoise Vermeylen, David Pelletier

**Affiliations:** 1 PO Box 54, Lemont, PA 16851 USA; 2 Division of Nutritional Sciences, Cornell University, 120 Savage Hall, Ithaca, NY 14853 United States; 3 World Vision Haiti, Port-au-Prince, Haiti; 4 Faculty of Health Sciences, Université Quisqueya, 218 Avenue Jean Paul II, Port-au-Prince, Haiti; 5 Division of Nutritional Sciences, Cornell University, B19 Savage Hall, Ithaca, NY 14853 United States; 6 Division of Nutritional Sciences, Cornell University, 212 Savage Hall, Ithaca, NY 14853 United States

**Keywords:** Antenatal care, Quality of care, Mobile clinics, Community outreach

## Abstract

**Background:**

Antenatal care (ANC) is an important health service for women in developing countries, with numerous proven benefits. Global coverage of ANC has steadily increased over the past 30 years, in part due to increased community-based outreach. However, commensurate improvements in health outcomes such as reductions in the prevalence of maternal anemia and infants born small-for-gestational age have not been achieved, even with increased coverage, indicating that quality of care may be inadequate. Mobile clinics are one community-based strategy used to further improve coverage of ANC, but their quality of care delivery has rarely been evaluated.

**Methods:**

To determine the quality of care of ANC in central Haiti, we compared adherence to national guidelines between fixed and mobile clinics by performing direct observations of antenatal care consultations and exit interviews with recipients of care using a multi-stage random sampling procedure. Outcome variables were eight components of care, and women’s knowledge and perception of care quality.

**Results:**

There were significant differences in the predicted proportion or probability of recommended services for four of eight care components, including intake, laboratory examinations, infection control, and supplies, iron folic acid supplements and Tetanus Toxoid vaccine provided to women. These care components were more likely performed in fixed clinics, except for distribution of supplies, iron-folic acid supplements, and Tetanus Toxoid vaccine, more likely provided in mobile clinics. There were no differences between clinic type for the proportion of total physical exam procedures performed, health and communication messages delivered, provider communication or documentation. Women’s knowledge about educational topics was poor, but women perceived extremely high quality of care in both clinic models.

**Conclusions:**

Although adherence to guidelines differed by clinic type for half of the care components, both clinics had a low percentage of overall services delivered. Efforts to improve provider performance and quality are therefore needed in both models. Mobile clinics must deliver high-quality ANC to improve health and nutrition outcomes.

**Electronic supplementary material:**

The online version of this article (10.1186/s12884-017-1546-7) contains supplementary material, which is available to authorized users.

## Background

Antenatal care (ANC) is a package of services delivered to pregnant women that can contribute to multiple valued maternal, perinatal and fetal health outcomes. Despite the demonstrated efficacy of many of the interventions that are part of routine ANC and an increase in the proportion of pregnant women who seek ANC over the past 30 years, the prevalence of preventable and treatable ante-, peri- and post-natal conditions remains high [1–6]. In developing countries the prevalence of maternal anemia is over 40%, 27% of babies are small-for-gestational age and 40% of deaths in children under 5 (2.9 million) take place in the neonatal period [7–9]. Insufficient ANC coverage, inadequate number of ANC visits and poor-quality care contribute to these persistent, negative health outcomes [10–13].

Mobile clinics that travel to locations with limited or no access to health centers have the potential to increase ANC coverage. Community-based outreach such as mobile clinics are promoted in *The Lancet’s* various series on maternal and neonatal survival and maternal and child undernutrition as a potentially effective delivery strategy [5, 6, 14, 15]. Although there is strong evidence for the effectiveness of some community-based delivery platforms, documentation of the implementation and effectiveness of mobile clinics, in relation to maternal and neonatal outcomes, is weak [16, 17]. Mobile clinics are associated with earlier and increased initiation of ANC, but are inconclusive for pregnancy outcomes in the United States and in one Sub-Saharan African country [18–20].

Quality of health care and health provider performance are increasingly monitored in developing countries to improve accountability of providers and health programs, for impact evaluation and for policy-related research [21]. Quality of healthcare, including ANC, does not have a single definition or measure, and has therefore been operationalized in myriad ways [22–26]. A common assessment approach is the systems model developed by Donabedian [27], which assesses structure, process and outcomes of care, with the assumption of the interconnectedness of these three components.

At the time of this study, the World Health Organization (WHO) promoted the “focused antenatal care” (FANC) model of care, which included four ANC visits during pregnancy and content specific to the timing of these visits [28]. The adoption of FANC in Sub-Saharan African countries with weak health systems has encountered numerous problems, prohibiting its success. These include high staff turnover, low provider-to-patient ratio and lack of training, equipment and supplies [29–33]. Given the challenges of FANC implementation in low-income countries, it is not surprising that many countries have not adopted this model [34–36].

Haiti is one such country. ANC coverage in Haiti is high compared to similarly developed countries [37]. Nationally, 90% of women attended one ANC visit and 67% attended at least 4 visits. Sixty percent of pregnant women sought their first ANC visit between months 4–5 of pregnancy and 21% between months 6–7 [38]. ANC throughout Haiti is delivered through a patchwork of government, nongovernmental organizations (NGOs) and jointly-managed health clinics.

In the study reported here, we used a cross-sectional design to assess and compare the quality of antenatal care between fixed and mobile clinics, including educational outcomes and the perceived quality of ANC by pregnant women [27]. Based on a search of PubMed and the Cochrane Review Library, we believe this is the first study to comprehensively evaluate quality of care delivered in a community-based model with fixed health care centres in a low-income country.

## Methods

### Study setting

The study took place in the Central Plateau of Haiti, one of the poorest areas of the country [38]. According to the 2012 Demographic and Health Survey, 84% of women in this region stated that lack of money and 60% of women stated that distance to health care were obstacles to seeking health care when needed [38]. Thirteen of the 17 health centers in this region receive substantial external financial, logistical and/or human resource support from NGOs. This support reduces fees that women pay for ANC in the Central Plateau; care is often free with possible small fees to register at a clinic or for supplies, such as nutritional supplements, insecticide-treated bednets, water cleansing tablets and birth kits. This low cost of care is likely the reason that the Central Plateau has the highest rate of ANC attendance in the country (94%) [38]. Routine ANC is offered in dispensaries, health centers, and hospitals, with lower-skilled staff in dispensaries and higher-skilled staff primarily in hospitals. In addition to these fixed health facilities, a large multi-national NGO implemented mobile clinics as part of a 5 year Maternal and Child Health and Nutrition Program funded by USAID (Title II). This study was conducted in the communes where the mobile clinics operated, in 10 of the 12 communes of the Central Plateau.

### Program description

The mobile clinic program provided free pre- and post-natal care to women who qualified for the program (although women did pay a minimal fee for certain supplies, such as iron folic acid supplements). Each month, 130 clinics were held in locations with limited access to health services. These clinics were held at a predetermined place and time each month and staffed by 40 health professionals, ranging from auxiliary nurses to nurse-midwives. Additional components of the program included mother’s clubs, where age-appropriate health and nutrition lessons were taught using behavior change communication, rally posts, where growth monitoring and vaccines were provided for children under 5, and food aid distribution.

### Study design and selection of health centers and study participants

This study used a multi-stage random sampling procedure. First, we designed the sampling frame to include all of the fixed clinics in the 10 communes where the mobile clinic program operated. Next, for each fixed clinic, two mobile clinics within the catchment area of the fixed clinic were randomly selected, one from each stratum defined by distance to the fixed clinic: less than or greater than two hours walking distance from the fixed clinic.

Administrative permission was obtained from all health centers before the start of the study. Providers were eligible to participate in the study if provision of routine ANC was part of their daily activities and women were eligible if they presented for a routine ANC visit to a provider in the study and were over 17 years of age. Observers spent two days in each fixed health center and one day in each mobile clinic. If there were two ANC providers working simultaneously in a clinic, we attempted to deploy two observers, one for each provider. However, if there were two providers and only one observer present, the observer observed both, spending one-hour intervals with each. The observer performed oral informed consent with all health care providers and women prior to inclusion in the study.

A sub-sample of women whose consultations were observed was invited to participate in an exit survey. Every 30 min, the enumerator would approach the next woman who completed her ANC visit to ask if she would participate. In some cases, the enumerators shortened or lengthened the time interval of 30 min, depending on the “patient flow” for that clinic. However, they were instructed to maintain the same time interval throughout the day. This same enumerator performed oral consent for women prior to the exit survey. This research study was approved by the Cornell University Institutional Review Board and the Haitian Ministry of Health (MSPP) Public Health Bioethics Committee. Both IRB Committees approved of the use of oral consent for all participants.

### Assessment of quality of care

We operationalized Donabedian’s definition of quality of care by creating a rubric that contained eight care components, identified by the authors from a literature review of ANC recommendations and guidance published by WHO and Jhpiego [28, 39–41]. For each care component, we assessed process measures by observation of ANC consultations and immediate outcome measures of knowledge and perception of quality by surveying ANC recipients following their consultation. Observation and interview tools were developed using the Demographic and Health Survey Service Provider Assessment observation protocols and exit interview questionnaires as a guide [42]. There are three primary differences between Haitian ANC guidelines and FANC. Haitian guidelines: 1) recommend three, as opposed to four, visits, 2) define “women at risk” (or who is eligible for “basic care”, as defined by FANC) differently, and 3) suggest less education and counseling topics compared to FANC, and are not specific about the timing of delivery for educational and counseling messages (Additional file 1: Appendix 1 closely matches Haitian Guidelines) [28, 43]. The determination of quality in this paper is therefore reported by comparison of provider behavior to the Haitian national guidelines [43].

### Data collection and analysis

Three observers and five enumerators were trained in their respective roles during a two-week training that included two days of hands-on practice in a health center outside the study region. Data collection took place between June and August 2012. All research activities were performed in Haitian Creole. Study observers were instructed to sit in an unobtrusive spot of the consultation room and interact with the provider or client as little as possible. All observations were recorded on paper and entered into CSPro (US Census Bureau, Version 4.1) [44]. Exit surveys were performed predominantly with personal digital assistants (Hewlett Packard iPAQ Series with CSPro Software), using paper copies when these could not be charged. Study staff did not observe interactions between clients and other staff outside of the consultation room.

The sample size calculations for observations was based on a two-tailed test of sample means (fixed vs. mobile) for the physical exam portion of the consultation, with an effect size of 0.25 and assuming an alpha of 0.05 and power of 80%. This outcome was selected because it was hypothesized to have the least variation between providers and would therefore require the largest sample size to detect a difference between groups. Due to clustering of observations, we applied a design effect of 1.96, assuming 25 women per cluster and an intra-class correlation of 0.04, which was based on previous research of the mobile clinic program [45, 46]. Four hundred and ninety-six women in both fixed and mobile clinics were the desired group sizes, for a total sample of 992 women.

The observation instrument consisted of a 90 item checklist of eight distinct care components: 1) intake, 2) physical exam, 3) laboratory exam, 4) distribution of supplies (birth kits, insecticide-treated bednets, water cleansing tablets or multivitamins), iron-folic acid and Tetanus Toxoid vaccine, 5) educational messages and counseling, 6) health provider communication and interpersonal delivery, 7) infection prevention and control, and 8) documentation. Outcome indices were created for each care component, summing the process indicators (0 for no and 1 for yes) for each care component and converting these to the proportion of services provided to a woman. The denominator of five of the care components varied depending on the whether it was a woman’s first visit to the clinic, if it was her first pregnancy, the number of months pregnant, and if she was given antibiotics (Additional file 1: Appendix 1). Differences between fixed and mobile clinics for the quality of ANC for the eight care components were tested with random-effects models to account for clinics nested within sample clusters, with care providers included as a random effect to control for: a) multiple clients seen by each care provider, b) care providers serving multiple clinics and c) multiple care providers observed within each clinic. The outcome variables for intake, physical exam, education messages and counseling, health provider communication and interpersonal delivery were analyzed as continuous variables (xtmixed in Stata). The outcome variables for the lab exam, distribution of supplies, iron and folic acid (IFA) and Tetanus Toxoid, infection prevention and control, and documentation were converted to dichotomous variables, defined by presence of at least one of the services in that care component, due to the small number of variables in the indices and therefore limited variation (xtmelogit in Stata). We report the predicted proportion of recommended services provided to women for the four care components analyzed as continuous variables and odds ratios for the four care components variables analyzed as dichotomous variables. We tested if variables such as if a woman’s first visit to the clinic, if primigravid, month gestation, the order she was seen throughout the day or the observer affected the outcomes, but they did not, so we present unadjusted analyses. To measure the perception of quality of care we used the DHS exit survey questionnaire, which uses a Likert scale to assess 12 aspects of care [42] (Additional file 1: Appendix 3). Education and perception of quality of care variables were also analyzed using random effects models. Data analysis was performed in Stata (StataCorp, version 12.1) [47].

## Results

**Fig. 1:**
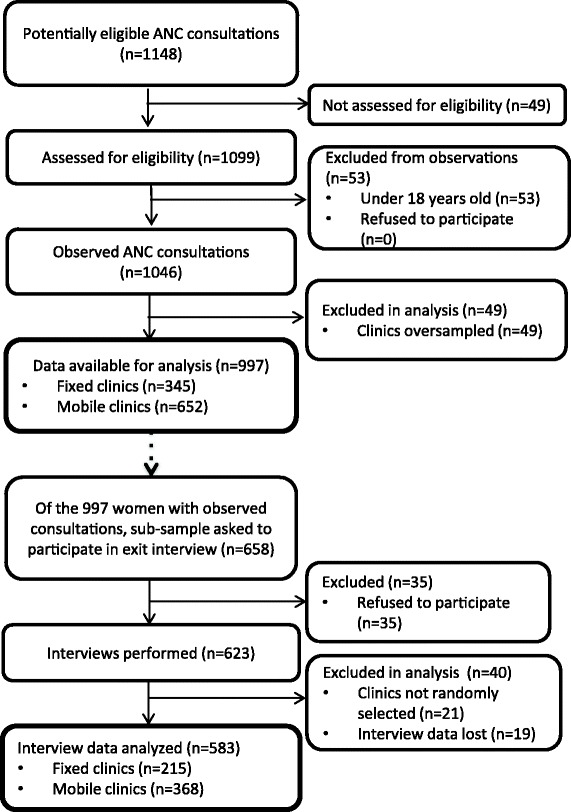
Flow Chart of Study Participant Recruitment, Participation and Analysis for ANC Observations and Interviews

Our final sample included fourteen fixed centers (four dispensaries, seven health centers and three hospitals) and 31 mobile clinics. Eleven fixed clinics were paired with two mobile clinics, as planned, three fixed clinics were matched with only one mobile clinic because in these cases there were no mobile clinics operating greater than 2 h from the fixed clinic, and three fixed clinics did not have any women present for ANC on the days enumerators were present. We retained all randomly selected mobile clinics matched with these health centers in the analysis. In these 31 clinics, we observed nine hundred and ninety seven observations, 345 from fixed clinics and 652 from mobile clinics (Fig. 1). Sixty-six percent of all women observed were asked to participate in the exit survey. Five percent of women declined to participate, with no differences in refusal rates between clinic type, leaving 585 interviews used in the final interview analyses (215 from fixed clinics and 368 from mobile clinics).

### Characteristics of ANC providers and recipients

Providers worked either in mobile or fixed clinics, but not both. The majority of providers in fixed clinics were observed once. In the mobile clinic program, ANC providers implement multiple mobile clinics, resulting in half of the mobile clinic providers being observed two or more times (Table 1). There were no differences in provider gender, training or number of consultations performed per day, nor were there differences in characteristics between women who were observed at fixed and mobile clinics (Table 2).

**Table 1:** Comparison of characteristics of antenatal care providers and clinics observed by clinic type (14 fixed and 31 mobile clinics)

	Fixed clinic providers (*n* = 24)	Mobile clinic providers (*n* = 19)	*p*-value
Number of times observed^a^			
1 time	21 (88%)	9 (47%)	<.01*
> 1 time	3 (13%)	10 (53%)	
Provider^a^			
Male	1 (4%)	2 (11%)	.42
Female	24 (96%)	16 (90%)	
Level of training^a^			
Doctor	1 (4%)	0 (0%)	.09
Nurse midwife	8 (33%)	1 (5%)	
Nurse	8 (33%)	8 (42%)	
Nurse assistant	7 (29%)	10 (53%)	
Mean number of ANC consultations performed per day per clinic (range)^b^	14.8 (2–37)	20.4 (3–55)	.12

Percent might not add to 100% due to rounding

*Statistically significant at *p* ≤ .05

^a^Significance testing performed using chi-squared test

^b^Significance testing performed using xtmixed adjusting for clinic, cluster and health care provider

**Table 2:** Characteristics of women with observed consultations and interviews by clinic type, accounting for clustering

	Fixed clinics	Mobile clinics	*p*-value
Women observed	*n* = 345	*n* = 651–652	
Primigravid	21.0%	20.0%	.67
First consultation	31.4%	36.0%	.44
Mean months pregnant if first visit to clinic^a^	4.82	5.26	.06
Mean months pregnant if not first visit to clinic^b^	6.63	6.59	.83
Women interviewed	*n* = 215	*n* = 367–368	
Primigravid	22.6%	18.9%	.40
First consultation	34.9%	34.3%	.74
Mean months pregnant if first visit to clinic^c^	4.92	5.18	.35
Mean months pregnant if not first visit to clinic^d^	6.35	6.60	.14

Predicted values and significance testing performed using xtmelogit or xtmixed adjusting for clinic, cluster and health care provider

Statistically significant at *p* ≤ .05

^a^ *n* = 106 and 252 for fixed and mobile clinics respectively

^b^ *n* = 231 and 387 for fixed and mobile clinics respectively

^c^ *n* = 75 and 126 for fixed and mobile clinics respectively

^d^ *n* = 140 and 241 for fixed and mobile clinics respectively

**Fig. 2:**
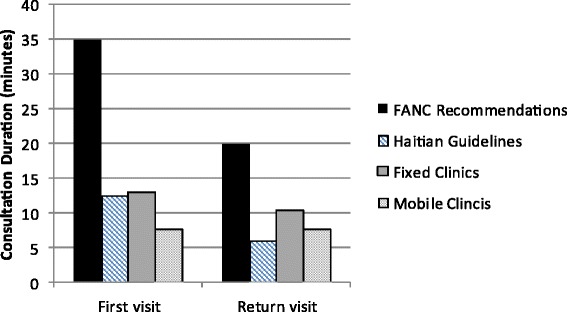
Consultation duration recommended by FANC and Haitian guidelines compared to observed practice in fixed and mobile clinics. FANC and Haitian Guidelines shown as mean of suggested range. *n* = 105 and 247 at fixed and mobile clinics respectively for first time visits. *n* = 225 and 376 at fixed and mobile clinics respectively for return visits

The duration of mobile clinic consultations was significantly shorter than fixed clinic consultations at both first (booking) and all subsequent, or follow-up, visits. First visits lasted on average 13.0 and 7.5 min in fixed and mobile clinics respectively and follow-up visits lasted 10.5 and 7.5 min in fixed and mobile clinics (Fig. 2).

### Inter-rater reliability

Inter-rater reliability was assessed by performing simultaneous observations with the head researcher (EP). All observers had above a 95% percent of agreement with the head researcher, with an average kappa statistic of .92, .94 and .94 for the three observers. This extremely high level of agreement beyond chance is likely due in part to the skewedness of variables and lack of variation in provider behavior [48]. However, it was found that two questions (checking the woman’s conjunctiva and palpation of abdomen for fetal presentation) had poor reliability and were fully dropped from the analysis. A third question related to disposal of medical waste was partially dropped from the analysis due to the observers’ misunderstanding of the question. This was clarified and subsequent responses retained in the analysis.

### Care components (process measures)

We found significant differences between fixed and mobile clinics in the predicted proportion or probability of services delivered to pregnant women for four out of eight care components: 1) intake, 2) lab exams performed, 3) infection prevention and control measures, and 4) distribution of supplements, IFA and Tetanus Toxoid (Table 3). Services for these care components were delivered more frequently in fixed clinics, except for distribution of supplements, IFA and Tetanus Toxoid, which were delivered more frequently in mobile clinics. There were no differences found between clinic types for the percent of services delivered for 1) the physical exam, 2) educational messages and counseling, 3) health provider communication and interpersonal delivery or 4) documentation. The range of the predicted proportion or probability of recommended services that were provided to patients for six of eight care components was between 0.06 and 0.55 in both clinic models; only documentation of the visit and provision of supplies were consistently performed for the majority of women.

**Table 3:** Comparison of the predicted proportion and odds ratios of recommended services provided to patients for care components between fixed and mobile clinics

Care component		Fixed clinic		Mobile clinic		
Continuous variables	Total n	n (%)	Predicted proportion (95% CI)	n (%)	Predicted proportion (95% CI)	*p*-value
Intake	995	345 (34.7)	0.28 (0.24, 0.32)	650 (65.3)	0.23* (0.20, 0.27)	*p* = 0.05
Physical Exam	994	344 (34.6)	0.53 (0.48, 0.58)	650 (65.4)	0.50 (0.45, 0.54)	*p* = 0.23
Educational messages and counseling	991	344 (34.7)	0.13 (0.09, 0.16)	647 (65.3)	0.10 (0.07, 0.13)	*p* = 0.17
Health provider communication and interpersonal delivery	973	345 (35.5)	0.47 (0.43, 0.50)	628 (64.5)	0.44 (0.41, 0.47)	*p* = 0.21
Dichotomous variables	Total n	n (%)	Odds ratio (95% CI)	n (%)	Odds ratio (95% CI)	*p*-value
Laboratory Exam	364	109 (29.9)	1.00 (reference)	255 (70.1)	>0.01 (0.00, 0.03)**	*p* < 0.01
Supplies, iron-folic acid supplements and tetanus toxoid	997	345 (34.6)	1.00 (reference)	652 (65.4)	5.07 (1.06, 24.34)*	*p* = 0.04
Infection prevention and control	469	112 (23.9)	1.00 (reference)	357 (76.1)	0.04 (<0.01, 0.07)**	*p* < 0.01
Documentation	987	341 (34.5)	1.00 (reference)	646 (65.5)	2.14 (0.49, 9.30)	*p* = 0.31

All values are adjusted for clinic, cluster and health care provider using xtmelogit or xtmixed

*p ≤ .05, **p < .01

Although there were no differences between clinic models in the proportion of recommended services provided for the physical exam component, just over half of these were performed in fixed clinics and 49% in mobile clinics. When we disaggregated the data, certain services, such as taking of blood pressure and uterine height, were provided consistently by providers in both clinic models and other services, such as measuring height or checking the perineal area (recommended for the first visit only), were rarely performed in either clinic model (data not shown).

The mobile clinic program did not provide lab exams due to safety and logistical constraints, which explains the extremely low probability and large difference in receipt of lab exams between clinic models. Differences in infection prevention and control between clinic models were driven by the smaller proportion of both hand sanitization (counted if the patient was touched by the provider) and proper disposal of medical waste in mobile clinics. There were few instances of improper disposal (*n* = 30 in fixed and *n* = 6 in mobile clinics), most often gloves or gauze with bodily fluid being left in a container with no top. No cases of improper needle disposal were reported.

Referrals play an essential role in the continuum of care for pregnant women and community-based mobile clinics can be an important link between communities and the health care system. Due to the small number of women referred from mobile clinics, these data were analyzed separately by comparing actual practice to Haitian guidelines [43]. Only a fraction of women who should have been referred were, in fact, referred: 5% (21/420) of women with high blood pressure > 140 mmHg and >90 mmHg in any trimester), 8% (37/463) of women over the age of 35 and 11% (4/36) of women with greater than six pregnancies.

### Women’s knowledge and perceived quality of care

**Fig. 3:**
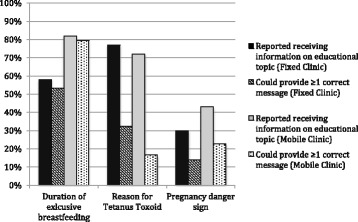
Reported receipt and knowledge of key educational messages, by clinic type. *n* = 212 and 260 at fixed and mobile clinics respectively

The Haitian Guidelines recommend that prenatal education include nine topics, including general nutrition and exclusive breastfeeding, the importance of vaccines and screening for underlying disease during pregnancy, birth planning and recognizing pregnancy and birth danger signs, and family planning. We asked women if they had received education on select, key topics at any time during their pregnancy and if they did, we probed to see if they could describe appropriate information for that topic (accepted responses listed in Additional file 1: Appendix 2) [43]. We found statistically significant differences in both the percent of women who said that they received and could provide one acceptable answer for two out of three key educational topics, the recommend duration of exclusive breastfeeding and pregnancy danger signs (Fig. 3). More women who attended mobile clinics were aware of the correct recommended duration of exclusive breastfeeding and of danger signs. In both clinic models, less than 35% of women could state why they were given a Tetanus Toxoid vaccine.

The perception of quality of care was similar for fixed and mobile clinics (data not shown). Two areas of care that received the lowest quality ratings in both clinic models were waiting time and the cleanliness of the clinic, although even these were deemed acceptable by 80% of women at both clinic models. Notably highly rated by women in both models was the treatment by the ANC provider and support staff and the cost of care.^1^

## Discussion

Mobile clinics have been proposed as a viable, alternative delivery model to increase ANC coverage, however, there is little prior evidence about quality of care in such clinics and how these compare to fixed clinics in the same regions. Our study compared fixed and mobile clinic quality in Central Haiti and identified differences in half of the care components studied (intake, receipt of lab exams, infection control and distribution of supplies, IFA and Tetanus Toxoid). Based on the relatively low percent of all possible services delivered in six of eight care components (excluding distribution of supplies, IFA and Tetanus Toxoid and documentation), we conclude that ANC quality was weak in both delivery models. Poorly delivered ANC is less likely to improve knowledge, behavioral or health outcomes, including detection of underlying health problems or pregnancy-related complications and proper management of danger signs, regardless of improvements in coverage.

### Care components (process measures)

A potential reason for the low adherence to the Haitian guidelines in both clinic models is the short duration of consultations, likely caused by the high volume of women in some clinics and large range of numbers of women who seek ANC: 2–37 per day in fixed clinics and 3–55 per day in mobile clinics. Additionally, some providers may have other responsibilities, such as performing post-natal or well-baby visits, but these were not counted or observed in our study. Fixed clinic consultations lasted on average five and three minutes longer for first and follow-up visits, respectively, than those in mobile clinics. However, it is not evident how this additional time was spent; women were asked a slightly higher proportion of the intake questions and lab exams (usually performed in a lab, not the provider) yet did not receive more time-consuming services, such as the physical exam or education and counseling. Possibly providers spent more time on counseling but if so, the effect of this was not seen in women’s knowledge assessed in the survey. Potential reasons for variation in consultation duration could include level of health care worker training (although this was not significant between clinic models) and experience or maternal factors, such as educational levels or socio-cultural issues. Short duration of consultations has been shown to limit adherence to ANC guidelines in other countries and likely prevents providers from making correct diagnoses and management plans [29].

Referrals were low for the three conditions studied in mobile clinics, most notably, for high blood pressure. High blood pressure and proteinuria are clinical criteria for identifying pre-eclampsia [49]. Although we were only able to assess referral for one of these criteria, 95% of women with high blood pressure were not referred to a higher-level center so that proteinuria could be measured. This is a grave missed opportunity for identifying women with hypertensive disorders, the cause of 22% of maternal deaths in Latin America and the Caribbean [50].

Two general trends emerge when we compare our study to other similar studies of ANC quality: 1) selective adherence to clinical guidelines and 2) better performance in the physical exam and health provider communication and interpersonal delivery components than education and counseling and intake. In our study and others, providers “pick and choose” services, providing some basic services to a high proportion of women and performing other services infrequently [51–53]. For example, across studies, measurement of blood pressure and uterine height, and listening to the fetal heart rate, are much more commonly performed in the physical exam than checking for edema or weighing patients [51–53]. This indicates that providers offer their version of “routine” care, which is incomplete according to clinical guidelines and not individualized to patient needs.

### Women’s knowledge and perceived quality of care

Group education sessions were the primary means of education, commonly performed by community health workers or nurses aides while women waited for their consultation. Yet, had this group education been effective, a higher proportion of women would have been able to provide at least one correct message per educational topic. Other studies have also documented low levels of education, counseling and knowledge in essential areas of birth preparedness and emergency readiness, indicating that this is a wide-spread problem of ANC programs [11, 53, 54].

Even with a low percent of clinical services being provided and poor education effectiveness, recipients of care perceived that they received high-quality care in both clinic types, which is slightly higher in mobile clinics. This paradox has been seen in other health contexts, where provider respect and politeness are deemed more important to patients than technical measures of care [55]. In this study, providers were consistently seen as treating patients politely and with respect, which likely mediated women’s perceived quality of care [56, 57]. Additionally, low consultation cost has been shown to increase patients’ perceptions of care quality in other developing countries [58].

Although we found that the quality of antenatal care differed between fixed and mobile clinics for half of the care components studied, differences in intake and supplies, IFA and Tetanus Toxoid were small and likely not clinically significant, while differences in receipt of lab exams and infection control were large and likely clinically meaningful. We therefore conclude that mobile clinics can provide similar quality of ANC as fixed clinics in the majority of care components studied. Differences in infection prevention and control measures could be improved, even within the current structure of the mobile clinics. The lack of lab exams offered in the mobile clinics is a potential structural weakness of the model, as carried out in this context. However this limitation does not negate its other potential benefits, and instead suggests the need for an integrated system of ANC with strong coordination of care between mobile and fixed clinics to optimize the continuum of care. For example, with strong communication and referral structures, women could attend mobile clinics for routine care much closer to home, travelling to distant, fixed sites for initial lab exams and when referred. This will only be effective, however, with system-level improvements in both models of care to reduce the number of consultations performed by providers per day and lengthening the duration of visits. This could be achieved by hiring more health staff to perform ANC or possibly through scheduling more mobile clinics to reduce the number of ANC consultations per day. Both options would require additional financial resources. In conjunction, institution- and provider-level interventions, including education, active supervision, audit and feedback and job aids, can improve provider adherence to guidelines and making correct diagnoses [29, 54, 59, 60].

This study took place in one of the poorest regions of Haiti, albeit one that receives substantial external support from NGOs. One example of this support is the strong supply chain for consumables, resulting in a high proportion of women who received IFA at their ANC visit. This finding is in contrast to many other studies in regions with less support, where supply chains function poorly [11, 51, 53]. Small scale and short-term mobile clinics are commonly employed in Haiti by NGOs to deliver general or highly-specialized health services (when foreign medical specialists visit, for example), for the purpose of increasing coverage in a country with weak infrastructure, a low provider to population ratio and poor access to health services [61]. However, the mobile clinic program described in this paper was unique in its large scale and duration (approximately 10 years, through two, five year grants). These results are therefore generalizable only to the Central Plateau of Haiti or similar regions where the health infrastructure receives external financial and technical support, yet poor health outcomes persist. Additionally, mobile clinics can be adapted to different contexts, and these adaptations will have varying impacts on quality.

There are some limitations of our study. This data was collected in 2012. Since that time the WHO released new, women-centered ANC guidelines [62]. We are unaware of any efforts to align national guidelines with these new guidelines, nor any large-scale efforts to improve ANC nationally. Additionally, we removed two important variables from the dataset, checking the woman’s conjunctiva and palpation of abdomen for fetal presentation due to poor reliability.

Observations are considered the “gold standard” for assessing quality of health care implementation, because they reduce potential risk of recall bias and poor or incomplete documentation [63, 64]. However, observations are limited by what can be visually or audibly assessed, and have the potential to alter client and patient behavior [63]. It is commonly assumed that observed health care providers are on their “best behavior” while observed. However, evidence has shown that providers quickly grow accustomed to being observed, minimizing this effect, especially if they do not know the study objective [24, 65].

We attempted to minimize any observer effect in the study design and analysis. First, neither the purpose of the study nor the study instruments were shared with care providers. Second, observers spent at least a full day with care providers, enough time for providers to resume their normal behaviors [65]. We tested if the order patients were seen each day altered the analyses, but it did not. Third, we explored the possibility that providers observed more than once might become used to being observed, resulting in a qualitative difference between being observed the first or future days, but did not find differences for any outcome. Given these results, and no reason to suspect a differential observation effect between fixed and mobile clinics, we believe that an observation effect did not substantially affect our conclusions.

It is possible that respondents in the exit survey were influenced by courtesy bias, compelling them to inflate their responses of perception quality of care. We attempted to minimize this effect by asking a mix of objective and subjective questions, as objective questions have been shown to be more comparable to results from community-based surveys of health care quality [58]. We found no difference in responses between these two types of questions.

Finally, observation of a single ANC visit, while informative about routine quality of care delivered in this region of Haiti, does not describe the sum of care received by women over the course of pregnancy. Our previous work on mobile clinics revealed that 54% of women who sought ANC at mobile clinics also sought care with another ANC provider [45]. It is possible, therefore, that by visiting multiple providers, women are, in fact, receiving more care than we were able to observe, although how the sum of this care compares to Haitian guidelines is not known.

## Conclusion

In this study we identified important gaps in ANC quality and potential reasons for these gaps in both fixed and mobile clinics. Although there were meaningful differences found between clinic models, particularly in the likelihood of lab exams provided and in infection control and prevention, the quality of ANC delivered through mobile clinics suffers from similar problems as fixed clinics in central Haiti, particularly in the areas of intake, the physical exam and education and counseling. Based on these findings, we believe that solutions to ameliorate these problems should be comparable between clinic models and address problems at multiple levels. With coverage of ANC increasing in developing countries, and the potential of mobile clinics to increase coverage even further, multiple means to improve quality of care will be essential to improve maternal and perinatal health.

## Additional file

Additional file 1: Appendix 1. Description of Contents for Eight Care Components. Appendix 2. Accepted Responses for Select Educational Topics. Appendix 3. Contents of Perceived Quality of Care Index. (DOCX 641 kb)

## Abbreviations

ANC: Antenatal care
FANC: Focused antenatal care
IFA: Iron and folic acid
NGO: Non-governmental organization
WHO: World Health Organization

## Footnotes

1 14% of women in fixed clinics and 69% of women in mobile clinics paid for their care. The median in both clinics was 25 Haitian goudes, approximately 0.55 USD.
