# Supplementary material for: Do mobile clinics provide high-quality antenatal care? A comparison of care delivery, knowledge outcomes and perception of quality of care between fixed and mobile clinics in central Haiti
Source: BMC Pregnancy Childbirth. 2017 Oct 16;17:361. doi: 10.1186/s12884-017-1546-7 (PMC5644158; doi:10.1186/s12884-017-1546-7)
Supplement: Additional file 1: — Appendix 1. Description of Contents for Eight Care Components. Appendix 2. Accepted Responses for Select Educational Topics. Appendix 3. Contents of Perceived Quality of Care Index. (DOCX 641 kb) [file 12884_2017_1546_MOESM1_ESM.docx]

**Additional file 1**

**Appendix 1 – Description of contents for eight care components**

**Care Component 1: Intake**

**Care Component 2: Physical exam**

**Care Component 3: Lab Exam**

**Care Component 4:** **Distribution of supplies, Iron Folic Acid and Tetanus Toxoid**

**Care Component 5:** **Health education and counseling**

**Care Component 6:** **Health provider** **communication and interpersonal delivery**

**Care Component 7: Infection Prevention and Control**

**Care Component 8: Documentation**

**Appendix 2 – Accepted Responses for Select Educational Topics**

| **Topic** | **Accepted Responses** |
| --- | --- |
| Recommended duration of exclusive breastfeeding | 6 months |
| Reason for tetanus toxoid vaccine | Tetanus  For a disease or infection  For my health and the health of my baby |
| Pregnancy danger sign | Hemorrhage  Fever  Swollen hands or face  Fatigue/shortness of breath  Headache or dizziness  Convulsion  Do not feel movement of baby/decrease in movement |

**Appendix 3 – Contents of Perceived Quality of Care Index**

| **Number** | **Aspect of Perceived Quality** |
| --- | --- |
| 1. | Wait time |
| 2. | Ability to ask questions |
| 3. | Amount of information received from provider |
| 4. | Visual privacy of consultation |
| 5. | Auditory privacy of consultation |
| 6. | Availability of medicine/supplements |
| 7. | Clinic hours |
| 8. | Days of available service |
| 9. | Cleanliness |
| 10. | Treatment by care provider |
| 11. | Treatment by other clinic staff |
| 12. | Cost of consultation and treatment |
